# Editorial: New advances in bedside assessment and monitoring of acute respiratory failure patients

**DOI:** 10.3389/fmed.2023.1154289

**Published:** 2023-03-02

**Authors:** Giuseppe Accurso, Luigi Vetrugno, Paola Pierucci

**Affiliations:** ^1^Department of Anaesthesia, Intensive Care and Emergency, Policlinico Paolo Giaccone, Palermo, Italy; ^2^Department of Medical, Oral and Biotechnological Sciences University of Chieti-Pescara, Chieti, Italy; ^3^Cardiothoracic Department, Respiratory and Critical Care Unit Bari Policlinico University Hospital, Bari, Italy; ^4^Section of Respiratory Diseases, Department of Basic Medical Science Neuroscience and Sense Organs, University of Bari “Aldo Moro”, Bari, Italy

**Keywords:** Central Venous Pressure, ARDS, hemodynamics, ultrasound elastography, Ventilator Induced Lung Injury (VILI), acute respiratory failure (ARF), extra corporeal membrane oxygenation (ECMO)

Four manuscripts were selected in the Research Topic titled “*New advances in bedside assessment and monitoring of acute respiratory failure patients*.” This editorial will highlight their major strengths and messages.

In the first manuscript, Tang et al. evaluated the outcomes of early using of Central Venous Pressure (CVP) in ARDS patients at ICU admission. They used retrospective info from the medical Information Mart for Intensive Care IV (MIMIC-IV) database. Their primary outcome was the 28-day mortality cut off (Tang et al.). Modern ICUs use invasive and non-invasive cardiac output measurements and the dynamic parameters of fluid responsiveness, such as stroke volume (SV), stroke volume variation (SVV), and pulse pressure variation (PPV), following the concept of functional hemodynamic monitoring to give fluids (1, 2). In patients with ARDS the measurement of EVLW with PICCO or EV 1000 are used, and the B-lines evaluation through Lung Ultra-Sound can be very useful (3–5). There is evidence against using CVP as a static hemodynamic index in critically ill patients, as CVP can't predict fluid responsiveness. Nevertheless, 45.6% of patients at risk for ARDS were in the CVP-group and showed shorter ICU stay, lower in-hospital mortality, higher fluid on day1 and better blood lactate clearance than those in the no-CVP- group (*p* < 0.001). Multivariate logistic regression confirms this trend (OR: 0.49; 95% CI: 0.42–0.57; *p* < 0.001), while the incidence of Acute Kidney Injury (AKI) was comparable between the two groups. Among the study limitations the patients in the CVP group showed lower severity of illness scores and fewer comorbidities, suggesting less impairment of overall function. The authors concluded that the association found does not necessarily imply causation and further prospective studies will be required to confirm whether CVP use is useful in ARDS patients.

In the second paper, Girard et al. measured the regional pleural strain related to invasive mechanical ventilation using ultrasound elastography. This is order to non-invasively evaluate the Ventilator Induced Lung Injury (VILI) and to measure if the US elastography would correlate with varying tidal volumes (Girard et al.). Pleural and diaphragmatic measurements are valuable tools used to indirectly check the lung parenchyma for consolidations and strength during mechanical ventilation and weaning off it (6, 7). Ultrasound radiofrequency speckle tracking allowed the computing of various pleural translation, strain and shear components. In this study, patients randomly received tidal volumes of 6, 8, 10, and 12 mL.kg 1 after anesthesia induction, while pleural ultrasound cine loops were acquired at 4 standardized locations. The authors screened 6 elastography parameters (lateral translation, complete lateral translation, lateral strain, absolute lateral strain, absolute lateral shear and Von Mises Strain) to identify those with the best dose response with tidal volumes using linear mixed effect models. This was possible in 90.7% of ultrasound cine loops. Intraobserver and interobserver measurements demonstrated high consistency and reliability. The authors found that measuring regional pleural strain by ultrasound elastography is feasible and reliable, however the pleural strain is not precisely equal to the pulmonary strain, and the sample size of 10 patients was small. Further larger studies will be need to confirm these results.

In the third manuscript, Chu et al. assessed the accuracy of diaphragmatic ultrasonography (DUS) in predicting the development of respiratory failure (RF) in patients with Community-acquired-pneumonia (CAP) and its feasibility and benefit in emergency department (ED) (8). In particular, they aim to demonstrate that diaphragmatic ultrasonography of diaphragm thickening fraction (DTF) in patients with CAP may be a reliable and accurate risk stratification tool to predict the development of RF. Despite the small sample (50 patients), the authors found that was an independent predictor of RF (69.23% sensitivity and 83.78% specificity) in patients with CAP, while diaphragmatic excursion (DE) was not. The authors calculated the thickening fraction as the maximal DT (assessed using a linear probe) during inspiration (Tdi, pi) minus the DT at end-expiration (Tdi, ee) divided by the Tdi, ee and multiplied by 100. The evaluation is very operator dependent. Even though the short training time required to obtain reliable measurements makes it ideal for the ED setting, experience is essential as small errors could lead to false-positive or negative. This study enhances early identification of patients with CAP at risk of rapid deterioration *via* point-of-care. They used a simple, low-cost tool easily accessible at bedside: ultrasonography (9, 10). The authors suggests that patients with DTF > 23.95% may be considered for outpatient management. These results in small numbers needs to be considered with caution and reinforced in larger studies.

Finally, the last study describes an upgrade of the V-V-system using an additional arterial return cannula (termed V-VA ECMO) to retain sufficient organ perfusion (Erlebach et al.). The V-VA ECMO provides both respiratory and hemodynamic support potentially representing a therapeutic option for patients with ARDS who develop secondary severe hemodynamic impairment. In this retrospective study among three ECMO centers, the authors aimed to describe the short and long-term outcomes of ARDS patients receiving V-V ECMO plus V-VA ECMO. The femoral-site for venous drainage and the jugular-site for venous return was the most frequent configuration of V-V ECMO. The femoral and subclavian artery cannulation was used in V-VA ECMO upgrade. The V-VA and V-V ECMO were successfully weaned in 47 (64%) and 40 (55%) patients. Thirty-five (48%) V-VA ECMO patients died during their ICU stay. An overall 2 year-mortality of 58% (39/67) was observed. In the final multivariable model, SOFA score >14 (HR 4.28; 95% CI: 1.55–11.80, *p* = 0.005) and lactate level [(HR 1.004; 95% CI: 1.000–1.008), *p* = 0.049] were significantly associated with 60-day ICU-mortality. The favorable ICU survival rate of 52% is remarkable, underling the message that in highly specialized centers, the upgrade of V-V ECMO patients suffering from ARDS to V-VA ECMO should not be rendered as futile *per se*.

## Author contributions

GA wrote the manuscript. PP and LV conceived the content, gave important intellectual contributions to the manuscript, and revised the draft critically. All authors contributed to manuscript revision, read, and approved the submitted version.
